# Equine Drug Transporters: A Mini-Review and Veterinary Perspective

**DOI:** 10.3390/pharmaceutics12111064

**Published:** 2020-11-08

**Authors:** Brielle Rosa

**Affiliations:** Department of Comparative Biology and Experimental Medicine, Faculty of Veterinary Medicine, University of Calgary, 3280 Hospital Drive NW, TRW 2D01, Calgary, Alberta T2N 4Z6, Canada; brielle.rosa@ucalgary.ca

**Keywords:** horse, drug transport, ATP-binding cassette, solute carrier protein, P-glycoprotein, breast cancer resistance protein, multidrug resistance protein, multidrug and toxin extrusion transporters, drug-drug interactions, veterinary medicine

## Abstract

Xenobiotic transport proteins play an important role in determining drug disposition and pharmacokinetics. Our understanding of the role of these important proteins in humans and pre-clinical animal species has increased substantially over the past few decades, and has had an important impact on human medicine; however, veterinary medicine has not benefitted from the same quantity of research into drug transporters in species of veterinary interest. Differences in transporter expression cause difficulties in extrapolation of drug pharmacokinetic parameters between species, and lack of knowledge of species-specific transporter distribution and function can lead to drug–drug interactions and adverse effects. Horses are one species in which little is known about drug transport and transporter protein expression. The purpose of this mini-review is to stimulate interest in equine drug transport proteins and comparative transporter physiology.

## 1. Introduction

The disposition and pharmacokinetics of many drugs are dependent on the activity of transport proteins located within cell membranes. These transporters move their substrates into and out of cells, and play a critical role in the ADME processes of absorption, distribution, metabolism, and elimination. While hundreds of transport proteins play critical roles in moving endogenous metabolic products throughout the body, only a small number of transporters bind and transport the drugs commonly used in human and veterinary medicine. Drug transporters fall into one of two transporter superfamilies: the solute carrier (SLC) superfamily, and the ATP-binding cassette (ABC) superfamily [1,2]. In general, these transporters share the ability to move structurally varied molecules across cell membranes. SLC drug transporters are usually, although not always, uptake transporters that facilitate the movement of drugs into cells, either from the blood, the extracellular fluid, or the external milieu. Drug transporters in the ABC superfamily are typically efflux transporters; they move drugs out of cells. This can include transport of drugs into the urine, bile, or gastrointestinal tract, or in the case of blood–tissue barrier sites such as the blood–brain barrier (BBB), can include transport of drugs out of tissue barrier cells and back into the bloodstream [3]. 

The SLC superfamily is a large and diverse grouping of membrane proteins that uses a variety of non-ATP-dependent processes to carry substrates across cell membranes. SLC transporters carry a wide array of substances, including amino acids, neurotransmitters, sugars, and certain pharmaceutical agents [4]. The specific SLC families that have been associated with drug disposition in humans and animals are: SLCO, which consists of organic anion transporting polypeptides (OATPs); SLC15, the family of peptide transporters (PEPTs); SLC22, which comprises organic cation and anion transporters (OCTs and OATs), and organic zwitterion/cation transporters (OCTNs); and SLC47, the multidrug and toxin extrusion transporters (MATEs) [4]. The OATPs, PEPTs, OCTs, OATs, and OCTNs play a critical role in the movement of charged organic compounds into cells. They are highly expressed in the small intestinal epithelium, liver, and kidney [5,6].

ABC transporters are ubiquitous transmembrane proteins that use ATP hydrolysis to move substances across cell membranes. In addition to their role in drug transport in eukaryotes they play an important role in prokaryotic drug resistance [7], and in multidrug resistant neoplasia [8]. Certain ABC transporters have commonly used human and veterinary drugs as their substrates. These transporters include: permeability glycoprotein (P-gp), encoded by the gene *ABCB1*; breast cancer resistance protein (BCRP), encoded by the gene *ABCG2*; and several of the multidrug resistance proteins (MRPs), which are encoded by *ABCC* genes [4]. Of these transporters, P-gp has been the most widely studied. The pharmacokinetic characteristics of many drugs depend on the interplay between the SLC and ABC transporters of which they are substrates, as well as other metabolic processes, and the physicochemical characteristics of the drug.

## 2. Organ-Specific Role of Drug Transporters in ADME Processes

The major organs that determine drug disposition and pharmacokinetics are the small intestine, liver, and kidneys. These three tissues regulate exchanges between the internal and external environment. Thus, it is unsurprising that drug transporters are highly expressed in these tissues. The specific effects of each of these organs on the disposition and pharmacokinetics of a specific drug will depend on the level of expression of transporters and drug metabolizing enzymes (DMEs) that act on that drug, and the site of transporter expression within their cell membranes. On the basolateral cell membrane, transporters regulate the uptake of drugs and other xenobiotics from the blood and their return to circulation. At apical sites, transporters absorb drugs and xenobiotics from, or excrete them into, the external environment. Apical sites in the liver, kidney, and small intestine are the canalicular, tubular, and luminal aspects of the cell surface, respectively [9]. Orally administered drugs are absorbed primarily in the small intestine. However, although the small intestine is a primary site of drug absorption, it is also a site of drug metabolism and excretion [10,11,12]. Illustrating this concept, peptide transporter 1 (PEPT1; gene name *SLC15A1* [4]) is an SLC uptake transporter in the human small intestine that is highly expressed in the apical membrane of enterocytes, and facilitates the transport of diverse substrates, such as β-lactams and angiotensin converting enzyme inhibitors, from the GI tract into the enterocyte, and thus into the organism [13,14,15]. Performing the opposite function are ABC transporters, also located in the apical membrane of enterocytes, which act to reduce absorption by returning drugs to the intestinal tract. These efflux transporters also work in synergy with cytochrome p450 (CYP) and other DMEs to facilitate drug metabolism in the intestinal epithelium and excretion of metabolites to the gastrointestinal tract, thus reducing distribution of the drug to the tissues [16]. This demonstrates the interplay between different transporters and DMEs. The absorption kinetics of any orally administered drug depend upon its physicochemical properties and the gastrointestinal transporters with which it interacts.

Drugs that have entered the body, whether by absorption from the intestinal tract or via parenteral administration, are generally subject to metabolism and subsequent excretion by the liver and/or kidneys. Drug metabolism involves oxidation and reduction reactions by CYP enzymes, and/or conjugation reactions catalyzed by transferase enzymes. The liver is considered the primary organ of drug metabolism, and hepatocytes express high levels of DMEs; however, DMEs are also present within the renal tubular epithelium and intestinal epithelium, as well as in other tissues [9]. In the liver, SLC uptake transporters are expressed on the basolateral side of hepatocytes and facilitate hepatocyte uptake of circulating drugs from the plasma [16,17]. Efflux of drugs from the liver into the biliary canaliculi is mediated by ABC transporters and by MATE1 expressed on the apical surface of hepatocytes [17,18]. In the kidney, uptake transporters on the basolateral surface of tubular epithelial cells work in coordination with efflux transporters on the luminal surface to facilitate movement of drug and drug metabolites from the blood into the urine [19]. The intestine has a dual function in regulating the absorption of orally administered drugs, and excreting certain drugs and xenobiotics from the systemic circulation [11,20]. Intestinal efflux transporters, in addition to their function in absorption regulation, can also excrete into the gastrointestinal tract circulating drug that has been distributed to the intestinal epithelium [20,21]. Thus, a drug can be excreted in the feces via both biliary and direct intestinal excretion. Collectively, the impact of transporter expression on drug disposition depends upon the types of transporters that interact with the drug, where they are expressed, and their level of expression.

## 3. Species Differences in Drug Transporter Expression: Importance in Veterinary Medicine

The majority of drug transport studies have been done using rodent or human tissues. These have shown that, although there is frequently a good correlation between species in transporter structure and substrates, there are also important species differences that can impact the success of clinical treatments used in veterinary medicine, and affect the utility of animals as pre-clinical models for humans [18]. For example, in cats, the ABC transporter, Bcrp, has amino acid variations at regions of its sequence that are highly conserved in other species. These differences are associated with a decrease in feline Bcrp function that, when fluoroquinolone antimicrobials are administered, results in retinal accumulation of phototoxic compounds leading to retinal degeneration and blindness [22]. It has also been suggested that the decreased functionality of Bcrp in cats may play a role in the increased susceptibility of their erythrocytes to hemolysis relative to those of other species, and in the toxicity of low-dose acetaminophen to cats [22]. Clearly species differences in drug transporters can have important clinical effects.

In addition to their influence on individual drug pharmacokinetics, transporters play an important role in drug–drug interactions (DDIs). Competition between substrates of the same transporter can affect the handling of either, or both, substrates. Some drugs act as inhibitors of specific drug transport proteins, impacting the uptake or efflux of substrates of the affected protein [16,23]. These interactions are also affected by species differences in transporter expression and activity. A recent in vitro study of DDIs made using cultured hepatocytes from humans, rats, dogs, and monkeys found sufficient inter-species variation to cause the authors to conclude that short-term cultured human hepatocytes were the best pre-clinical model to use to predict DDIs in between-liver uptake transporter-substrate drugs in humans [24].

In veterinary medicine, successful treatment of disease in the variety of animal species seen by veterinary practitioners relies on species-specific knowledge of appropriate therapeutic choices and dosing protocols. This requires understanding the specific factors that contribute to the disposition and pharmacokinetics of potential therapeutic drugs in the species being treated. In companion animals, particularly in dogs, there has been some significant progress in understanding the mechanisms of drug disposition and ADME processes. This is due to the combined efforts of researchers conducting veterinary and human pre-clinical studies. Dogs are commonly used as pre-clinical models of cancer [25], heart disease [26], and other disorders [27]. There is also a body of literature describing drug transport into the mammary gland of cattle because of the obvious economic and human health concerns related to this issue [28,29]. However, for the other domestic animal species commonly treated by veterinarians there have been few studies of drug transport. This has led to inter-species extrapolation of likely DDIs from human data, which is of questionable accuracy [24]. Physiologically-based pharmacokinetic (PBPK) modelling, and in vitro to in vivo extrapolation are gaining traction as methods of predicting drug pharmacokinetics and interactions, but accurate computer models require knowledge of the physiological parameters that influence drug handling in the body. This knowledge is lacking for most domestic animal species.

One particular species in which very little work on drug transporters has been done is the horse. As large herbivorous hindgut fermenters, horses have evolved to withstand the ingestion of photoactive compounds and potential dietary toxins in their forage. Their cecal microbes also produce large quantities of monoamines, which are absorbed, and may throughout the course of evolution have impacted their DMEs [30], as well as other metabolic pathways and related transport proteins. Clinically, horses respond differently to some drugs than other species; for example, oral absorption of many therapeutics is lower in horses than in humans and other species, possibly due to differences in intestinal uptake transporter expression [31]. This limits the clinical utility of many therapeutic options in horses. A recent study that examined the pharmacokinetics and oral bioavailability of apixaban, an anticoagulant drug that has been shown to be bioavailable in dogs, cats, rats, chimpanzees, and humans, found that a dose of apixaban equivalent to an 85% bioavailable dose in cats had 0% bioavailability in the horse [32]. This may be due to the fact that apixaban is a P-gp substrate [33], and the high levels of P-gp expressed in the equine small intestine [34] may reduce its absorption. Interestingly, the other species in which apixaban is known to have very low bioavailability is the rabbit [35], also a hindgut fermenter. However, despite the U.S. horse industry’s estimated economic impact of $122 billion dollars and 1.7 million jobs (https://www.horsecouncil.org/resources/economics/ accessed on 11 September 2020), there are few studies available that specifically examine drug transporter expression and function in the horse. The purpose of this mini-review is to summarize what information is available on equine drug transporters and to provide clinical correlations and highlight potential areas for future research.

## 4. Drug Transporter Expression and Localization in the Liver, Kidney, and Small Intestine of Horses

### 4.1. Transporter Mediated Drug Uptake

Understanding the species-specific role of uptake transporters in the disposition, metabolism, and excretion of any individual drug is very important for understanding the pharmacokinetics of that agent, and for the prediction of clinically important DDIs. Members of the SLC superfamily mediate the cellular uptake of drugs from both the external environment and the systemic circulation. Very few studies have examined uptake transport in horses, but many uptake transporters have been identified that influence drug disposition in humans. By facilitating the movement of their substrate drugs into human enterocytes, uptake transporters, such as PEPT1 (*SLC15A1*), impact drug absorption. By transporting drugs from the circulation into human hepatocytes, uptake transporters such as OATP1B1 (*SLCO1B1*), OATP1B3 (*SLCO1B3*), OAT2 (*SLC22A7*), OCT1 (*SLC22A1*), and OCTN1 (*SLC22A4*) and OCTN2 (*SLC22A5*), facilitate metabolism and clearance [17,36]. Similarly, transport of drugs into the cells of the human kidney by transporters such as OAT1 (*SLC22A6*), OAT3 (*SLC22A8*), OCT2 (*SLC22A2),* and OATP4C1 (*SLCO4C1*) enables renal clearance of these compounds by subsequent secretion from the renal tubular epithelium [37]. Although the transporters listed here have not been specifically investigated in the horse, their homologues likely play a role in equine xenobiotic disposition and transport.

In the human liver, the most abundant SLC transporter is OATP1B1, which has been implicated in numerous clinically important DDIs [38,39]. Expressed in the basolateral (sinusoidal) cell membrane of hepatocytes [40], OATP1B1 transports both endogenous and xenobiotic molecules into cells, facilitating hepatocellular uptake and then the subsequent metabolism/excretion of these agents [17,41]. Known substrates of OATP1B1 that are of particular interest in equine veterinary medicine include β-lactam antimicrobials, opioids, rifampin (also referred to as rifampicin), bile acids, and bilirubin; rifampicin is also an OATP1B1 inhibitor [4]. The closest homologue to human OATP1B1 that exists in horses is Oatp1b4 (gene name *Slco1b3*); this protein shares 70.04% sequence homology with human OATP1B1 (protein BLAST; https://blast.ncbi.nlm.nih.gov/Blast.cgi?PAGE = Proteins), but has been named based on genome annotation, and has not been experimentally shown to have similar function [42]. Equine Oatp1b4 also shares 71.15% sequence homology with human OATP1B3. Since Oatp1B4 is the equine homologue of both OATP1B1 and OATP1B3 in humans, it is likely that it plays a similar role in bringing drugs into the liver. Dogs [43] and rodents [44,45] both have a single Oatp1b family transporter that is structurally similar to OATP1B1 and OATP1B3 in humans. In dogs, the single Oatp1b4 has been shown to be similar, but not identical, in function to its OATP1B1 and OATP1B3 orthologs that occur in humans and other primates [43]. In general, across species, the liver is the primary organ of drug metabolism, and transporter-mediated hepatic uptake of a circulating drug impacts its metabolism and plasma clearance [17]. In humans, OATP1B1 and OATP1B3 transport are associated with DDIs involving statins, cyclosporine, and other commonly used drugs [46]. Inhibition of hepatic uptake transporters can increase circulating plasma concentrations of a drug, as occurs in human patients when certain statins are co-administered with OATP inhibitors [47,48].

An OATP transporter with a much broader tissue distribution than OATP1B1 and OATP1B3 is OATP2B1/Oatp2b1 (*SLCO2B1/Slco2b1*). This transport protein is expressed in the liver, kidney, heart, brain, and placenta of humans [49], and in horses its mRNA has also been demonstrated in those tissues [50]. It is also expressed at a constant level throughout the entire length of the human intestine [51], but its expression in the equine intestine has not been assessed. It displays pH-dependent substrate specificity, and has been considered a player in the uptake of exogenous substrates from the intestinal lumen [52]. However, recent work indicates that its distribution is basolateral rather than apical [53], and its uniform distribution along the length of the intestine also suggests a function other than luminal uptake [51]. This basolateral distribution in the enterocyte is consistent with its role as a facilitator of biliary elimination of conjugated substrates, as apical distribution distal to the duodenal papilla would result in reabsorption of excreted compounds [53]. In the horse, Oatp2b1 is also moderately expressed in the ovaries, where it may play a role in the follicular cycle [50].

The results of a study examining the pharmacokinetics of the antihistamine fexofenadine, a P-gp substrate [54], in horses suggest that OATPs may play a role in equine intestinal drug absorption. In this study, horses were administered fexofenadine orally and intravenously, both with and without pretreatment with the P-gp inhibitor, ivermectin. Ivermectin pretreatment did not affect the pharmacokinetics of intravenous fexofenadine, but reduced the bioavailability of oral fexofenadine from an already poor 2.6% to only 1.5% [55]. This was unexpected, since inhibition of intestinal P-gp by ivermectin would have been expected to increase fexofenadine absorption. However, it was theorized by the authors that perhaps ivermectin inhibited an OATP uptake transporter. Fexofenadine is an OATP1A2 substrate [56], and OATP inhibition has been shown to decrease fexofenadine bioavailability in humans [57] and rats [58].

The fexofenadine example highlights the complexity of DDIs involving multiple drug transporters. Similar interactions could occur in the kidney, where uptake and efflux transporters work in cooperation to move drugs and metabolites out of circulation and into the renal tubular ultrafiltrate for excretion in the urine [39,59]. Impaired uptake into, or efflux from, the renal tubular epithelial cells can result in decreased elimination and prolonged drug exposure [37]. This has been exploited since the 1950s, when it was discovered that concomitant administration of probenecid increased serum penicillin levels and was useful in treating penicillin-resistant infections [60]. Coadministration of drugs that inhibit renal efflux but not uptake transporters can lead to accumulation of nephrotoxic levels of the drug in the renal epithelial cells, as happened in a human patient who was coadministered diclofenac and tenofovir [61]. A detailed understanding of drug-transporter interactions is needed to avoid this type of DDI. Horses are known to experience drug-related nephrotoxicities. These are most often associated with aminoglycoside antimicrobials or non-steroidal anti-inflammatory drugs, such as phenylbutazone [62], but the role of drug transport in drug-associated nephrotoxicity in the horse not currently understood. Recently, it has been suggested that overexpression of OCT2 (*SLC22A2*) may contribute to the increased accumulation of the aminoglycoside gentamicin in the kidneys of obese animals and humans [63]. This provides an indication that drug transport and transporter expression may also be involved in the physiological processes underlying the nephrotoxicity of these drugs in horses. Reabsorption of drugs from the tubular lumen can also influence renal clearance. Uptake transporters are located in both the apical and basolateral membranes of renal tubular epithelial cells [37]. OCTNs in the apical membrane act as bidirectional transporters, and play a role in both uptake and efflux [6].

### 4.2. Transporter-Mediated Drug Efflux

Efflux of drugs from the liver and other organs is mediated primarily by ABC transporters. These ATP-dependent transport proteins are ubiquitously distributed throughout the body, but in the liver are primarily located on the apical (canalicular) surface of hepatocytes [17]. ABC efflux transporters in the human liver, kidney, and small intestine include P-gp (*ABCB1*), BCRP (*ABCG2*), and MRPs (*ABCC*s) [16,17]. Due to their utilization of ATP to provide energy for transport, they are able to move their substrates against concentration gradients [64].

#### 4.2.1. P-glycoprotein (P-gp/*ABCB1*)

P-gp is a highly conserved ABC transport protein that is present in nearly all tissues [17]. It has been the most widely studied of the drug transporters across all species. It is a non-specific efflux pump with a wide array of chemically unrelated substrates [16,65]; those of particular importance to equine veterinary medicine include: ranitidine [66], dexamethasone [67], ivermectin [67,68], opioids [69], tetracycline [70], rifampin [71], macrolides [72,73,74], and certain β-lactam antimicrobials [75]. Notably, none of these compounds have been specifically demonstrated to be substrates of equine P-gp; evidence of their status as P-gp substrates comes primarily from work examining human and rodent P-gp transport. However, while species-specific differences in P-gp substrate specificity do exist [76,77], they tend to be rare [18]. Equine and human P-gp are similar in structure, with 90.3% homology predicted, based on computer modeling/genome annotation [42,78], and it is likely that they share many of the same substrates. In addition to its wide array of substrates, P-gp activity is also subject to modulation by many compounds, including common therapeutic drugs and dietary components [79]. Owing to this, P-gp is associated with numerous DDIs in humans and in veterinary species [65]. Polymorphisms of P-gp are associated with alterations in drug disposition and kinetics [79]. The most prominent example of a polymorphism-associated DDI in veterinary medicine is the severe neurotoxicity after ivermectin administration that occurs in dogs with an *Abcb1* polymorphism, resulting in a dysfunctional P-gp. Normally, P-gp plays an important role in limiting the intestinal permeation of ivermectin, as well as limiting its movement through the blood–brain barrier [80]. In affected dogs, a four base pair polymorphism results in a truncated and non-functional P-gp that allows greater uptake of ivermectin, and distribution to the central nervous system, resulting in neurotoxicity, seizures, and sometimes death of the affected animals [81]. The non-functional P-gp impacts the disposition of other veterinary drugs as well, and may influence treatment decisions for affected animals. This polymorphism occurs more frequently in herding-breed dogs than in other breeds, and genetic testing is often recommended for individuals belonging to predisposed breeds or for mixed breed dogs prior to extra-label high dose use of drugs that are P-gp substrates [80,81].

In horses, P-gp has been shown to be highly expressed in the liver, and has been localized to the apical membranes of hepatocytes by immunohistochemistry [78]. In the equine intestine, Western blotting demonstrated a high level of P-gp expression in the enterocytes of the mid-duodenum and ileum, with a lesser degree of expression in the other regions of the small intestine [78]. The relative levels of P-gp expression in the liver versus small intestine of the horse, as determined by ChemiDoc screening of standard Western blots, were reportedly similar; however, no attempt at statistical analysis of the quantification has been made [78]. In humans, expression of P-gp is seven times greater in the small intestinal enterocytes than in the liver, suggesting that it plays a more substantial role in xenobiotic efflux and limiting absorption at the small intestine than it does in biliary drug excretion [17]. Whether this is also the case in horses remains to be proven, although one study using equine jejunal mucosa ex vivo did show that P-gp limits the absorption of the opioid, methadone. When intestinal explants in Ussing chambers were exposed to verapamil, a potent P-gp inhibitor, methadone flux across the intestinal mucosa significantly increased [34]. The same study also demonstrated that P-gp expression in the equine small intestine was greatest in the apical membrane of the cells at the tips of the intestinal villi, and was highly variable between individuals [34]. It is worth noting that the impact of P-gp on small intestinal absorption of its substrates is variable and that the transport capacity of P-gp can be saturated [82]. Thus, its effect on small intestinal absorption tends to be greatest for those substrates given at low doses, or those that are absorbed over a prolonged period of time [82].

Only one study has examined P-gp expression in the equine kidney. In that study, immunohistochemistry of the equine kidney showed that P-gp was strongly expressed on both the apical and basolateral sides of the renal epithelial cells lining the loop of Henle, and strongly to moderately expressed on the apical sides of the proximal and distal renal tubular epithelial cells, while being expressed to a much lesser extent on the basolateral and intercellular surfaces of the tubular epithelial cells [78]. In the horse, P-gp mRNA expression did not correlate with P-gp levels in the intestine; this is similar to the what has been reported in humans, and considerable individual variation in P-gp expression has been shown in people [83]. Post-transcriptional regulation of P-gp protein levels by micro RNAs has been demonstrated, and may impact individual variation in P-gp expression and function [84], as well as multidrug resistance in certain cancers [85].

Many drugs and dietary chemicals have also been shown to affect P-gp expression [1,65]. This responsiveness to environmental influences likely underlies the implication of P-gp in numerous DDIs. The role of P-gp in DDIs, and in multidrug resistant neoplasia, has primarily been studied in humans and other non-equids. However, P-gp-related DDIs have also been demonstrated in horses. A very clinically relevant example from equine veterinary practice is the interaction between rifampin and macrolide antimicrobials, drugs which are commonly used in combination for the treatment of *Rhodococcus equi* infection. *R. equi* is an intracellular coccobacillus that causes pneumonia often associated with pulmonary abscessation in foals [86]. Although the majority of foals will recover without antimicrobial treatment, the current standard of care is to treat severely affected foals with the combination of rifampin and a macrolide antimicrobial, historically erythromycin, and more recently clarithromycin or azithromycin [86,87]. In 2011, Peters et al. demonstrated that chronic oral coadministration of clarithromycin and rifampin to foals reduces the bioavailability of clarithromycin by more than 90% [88]. In subsequent work, chronic rifampin treatment was shown to cause upregulation of P-gp in equine enterocytes; this reduced intestinal absorption of clarithromycin, and resulted in the decrease in the clarithromycin bioavailability previously reported [89]. Similar studies with azithromycin and rifampin in foals have not been conducted, but azithromycin has been shown to be a P-gp substrate in humans [73], and the DDI is likely to be similar. However, in spite of the negative impact of rifampin on the bioavailability of clarithromycin, the coadministration of these drugs (or a similar macrolide, such as azithromycin, with rifampin) is still recommended at this time [90]. This is because the combination has been shown to decrease the likelihood of resistance development, has some (limited) evidence of efficacy in vivo [91,92], and coadministration with rifampin also increases the concentration of clarithromycin in the epithelial lining fluid of the bronchioles, possibly through effects on pulmonary OATP transporters [89], resulting in it remaining at levels higher than the MIC90 of most *R. equi* isolates ( > 0.06 µg/mL) despite its decreased bioavailability [93,94]. Interestingly, macrolides have been shown to be P-gp inhibitors in vitro and via DDIs in human patients [23], but when given concomitantly with rifampin the upregulatory effect of rifampin on P-gp appears to take precedence.

Another drug that interacts with P-gp in a clinically important way is the anthelmintic, ivermectin. This drug is used extremely commonly in horses (and other domestic animals) to treat endo- and ectoparasite infestations [95], and has been shown to be both a substrate and potent inhibitor of P-gp [96]. Ivermectin is such an effective inhibitor of P-gp that it has been used in several pharmacokinetic studies to evaluate the DDIs between it and other P-gp substrates [55,97]. In a study investigating the pharmacokinetics of the antihistamine cetirizine in horses with and without ivermectin pretreatment, the authors found that the area under the curve (AUC) of cetirizine was greater when it was administered 12 h after ivermectin pre-treatment. They postulated several potential mechanisms that may have caused this interaction, but the most likely seems to be inhibition of renal P-gp, resulting in decreased excretion in the urine. In humans, cetirizine undergoes very little metabolism and is primarily excreted unchanged by the kidney, through a combination of glomerular diffusion and active tubular transport [98]. As a substrate of P-gp, inhibition of renal P-gp by ivermectin would decrease excretion of cetirizine, increasing the AUC. This demonstrates the potential for ivermectin-associated DDIs; however, specific studies evaluating the interactions of ivermectin with many of the drugs prescribed by equine veterinary practitioners have not been performed.

#### 4.2.2. Breast Cancer Resistance Protein (BCRP/*ABCG2*)

BCRP is another efflux transporter with a broad distribution and the ability to bind many chemically diverse substrates. There is substantial overlap between the substrates of BCRP and those of P-gp [99]. BCRP is especially important in the mammary gland and placenta of many species but it is also expressed in the liver, kidney, and intestine. In humans, BCRP has been suggested to be more important in the intestine and kidney than in the liver [17]. This may be the case in other species as well; in ruminants the small intestine and mammary gland express the highest levels of Bcrp, with the liver also expressing high amounts, and moderate expression in the lung, kidney, and colon [28]. Bcrp is upregulated by lactation, as evidenced by its 5−10 fold greater expression in the lactating mammary gland of ruminants than in the non-lactating mammary gland, and Bcrp function is an intrinsic determinant of drug residues in milk [28]. Human and horse BCRP/Bcrp are 86% homologous, but unlike in the human liver, where BCRP is expressed predominantly on hepatocyte canalicular membranes, the only study to have investigated Bcrp localization in the horse liver localized it not only to the canalicular membranes of hepatocytes, but also within the cytoplasm of hepatocytes in the peripheral parts of the liver lobules [100]. In ruminants, Bcrp has been found within the canalicular membrane of hepatocytes [28], and intracytoplasmic Bcrp localization has not been seen in these species, nor in dogs [101] or mice [102]. The intracytoplasmic localization of Bcrp in horses needs to be confirmed as it has only been demonstrated once in this species and has not been shown in healthy hepatocytes from any other species. However, if confirmed, it raises interesting questions regarding the role of Bcrp in the horse liver. Atypical Bcrp expression on the basolateral membrane of hepatocytes has been associated with liver disease in humans [103]. In rats, hepatic oval cells (progenitor cells capable of hepatocyte regeneration) are present in the peripheral hepatic lobules, and express Bcrp [104]. It has been proposed that atypical Bcrp expression may occur to protect the hepatocytes against damage/toxicity [100], and certainly, increased proliferation of Bcrp-expressing oval cells in the liver may occur in response to liver damage. The diet of the horse is rich in phytochemicals, many of which have toxic potential. Liver injury associated with diet is common in equids, and elevation of serum enzymes indicative of hepatocellular injury is frequently seen, even in apparently healthy horses [105]. It may be that the unusual intracytoplasmic expression of Bcrp seen in the horse is an adaptive response to hepatotoxic compounds in their diet. However, further research is needed on this topic.

In the equine small intestine, Bcrp is strongly expressed on the apical surface of enterocytes and diffusely throughout the cells of the serous acini of the Brunner’s glands in the submucosa of the duodenum and proximal jejunum. It is also expressed in the immune cells within the lamina propria of the cecum and colon [100]. In the kidney of the horse, immunohistochemistry revealed strong BCRP expression on all aspects of the epithelial cells lining the loop of Henle, and on the apical surface of the cells lining the distal convoluted tubules and collecting ducts, with weaker immunoreactivity evident on the basolateral aspect of these cells. Bcrp has also been identified in the endothelial cells in both the liver and kidney [100]. As is the case for P-gp, mRNA and protein expression of Bcrp are not correlated [100].

BCRP polymorphisms occur commonly in humans [106], and individual BCRP phenotype is a major determinant of response to drug therapy [99]. The frequency and type of BCRP polymorphisms differ between races, and it has been suggested that ethnic variation in the incidences of adverse events associated with BCRP-substrate drugs may be due to altered BCRP functionality [106]. Species-specific differences are also evident, such as the reduction in Bcrp function seen in cats that was described earlier in this article. No attempt has yet been made to determine if there are Bcrp polymorphisms in horses, and if these have any breed association; the clinical perception among many veterinarians is that there are differences among horse breeds in response to certain medications, especially sedatives that have rapidly observable effects (personal observation), but the actual extent of any breed differences in drug disposition or pharmacokinetics remains to be discovered [30].

#### 4.2.3. Multidrug Resistance Proteins 1–6 (MRP1—MRP6/*ABCC1—ABCC6*)

The MRPs are a nine-member family in the ABC superfamily of transporters; MRPs 1–6 are the most well characterized, in terms of their role in drug disposition. Most cells express one or several MRPs and, like the other efflux transporters discussed in this review, they have been associated with drug resistance in tumor cells [107]. Data from humans and rodents show that, in the liver, MRPs 3, 4, and 6 are strongly expressed on the basolateral surface of hepatocytes, and transport their substrates from the intracellular environment to the blood, while MRP2 is located on the canalicular membrane of hepatocytes, and effluxes its substrates to the bile [17]. MRP2 plays an important role in the excretion of glucuronidated bilirubin into the biliary tract [108], and is involved in the efflux of some drug conjugates [109]. MRP1 is expressed at low levels in the liver but is strongly expressed, along with MRP 2 and 6, in the kidney [16]. MRPs 3 and 6 are present on the basolateral surface of enterocytes in the small intestine, and MRP2 is present on the apical surface [16]. In general, the MRPs transport drug metabolic products of phase II metabolism.

In horses, only the expression of Mrp1 and Mrp2 has been investigated, and that has been reported in just one study [100]. In that study, Mrp1 was expressed in the equine liver and kidney at levels too low to detect by immunohistochemistry [100], thus its localization on the equine hepatocyte and renal tubular epithelium has not been reported. Mrp2 was expressed in the apical membrane of hepatocytes, in hepatic and renal arteriolar endothelial cells, at both the apical and basolateral surfaces of the epithelial cells lining the loop of Henle, and, to a lesser extent, the collecting ducts [100]. In the equine intestine, the distribution and extent of expression of Mrp1 and Mrp2 were also markedly different. Mrp2 was present on the apical surface of small intestinal enterocytes at the tips of the villi, while Mrp1 was intracytoplasmic in the enterocytes of the cecum and colon but localized to the apical side of the nucleus. Mrp1 was also present within the cytoplasm of the cells of the serous acini of the Brunner’s glands [100]. These findings suggest that ADME processes are not greatly influenced by Mrp1 in horses, but that Mrp2 may help to limit drug absorption in the intestine, and may play a role in renal drug/metabolite excretion. It is likely that the other Mrps also play a role in drug disposition in the horse; however, there are currently no data available from the horse on this topic.

**Figure 1:**
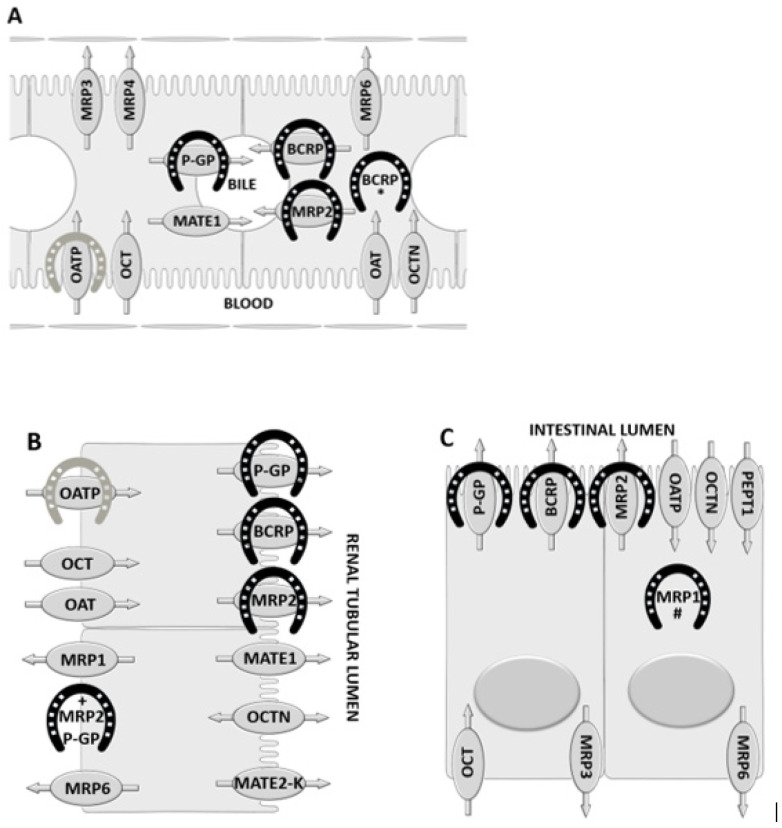
Schematic diagrams showing the localization of some of the major drug transporters in: (**A**) hepatocytes, (**B**) renal tubular epithelial cells, and (**C**) enterocytes. The proteins shown inside ovals have been identified in humans and/or rodents. Black horseshoes indicate transport proteins that have been described in horses. The light grey horseshoe indicates that, of the OATP family transporters, only Oatp2b1 has been specifically identified at the indicated locations in horses. * Breast cancer resistance protein (BCRP) has been identified in the cytoplasm of hepatocytes only in horses. + MRP2 and P-gp have been described in both the apical and basolateral membranes of renal tubular epithelial cells in horses. # MRP1 has been identified in the cytoplasm of enterocytes of the cecum and colon in horses.

A summary of current knowledge of the localization of drug transport proteins in hepatocytes, renal tubular epithelial cells, and enterocytes of horses and humans/rodents is shown in Figure 1. Table 1 provides information on the drug transport proteins that have been identified specifically in horses. 

#### 4.2.4. Other Efflux Transporters

There are other efflux transporters, in addition to the ones already discussed in detail in this article, that may also play a role in equine drug ADME processes. However, there are no data on their expression in the horse. These include MATE1, MATE2-K, and the bile salt export pump (BSEP). MATEs 1 and 2-K are SLC efflux drug transporters that are involved in the excretion of organic cations by the kidney (both) and liver (MATE1 only) [110]. In humans [59], primates, and rodents [19], MATEs are highly expressed in the kidney on the apical (luminal) membrane of renal epithelial cells, and renal excretion of organic cations is considered their major physiological function. In humans, the protein expression of MATE1 in the kidney has been weakly [59], and strongly [19], correlated with the expression of OCT2, an organic cation uptake transporter. MATE transporters have been implicated in DDIs in humans [111], but this has not been well-studied in veterinary species. Their relative importance in the efflux of drugs and toxins from the equine kidney and liver is currently unknown.

The BSEP is an ABC transporter that is expressed solely on the apical membrane of hepatocytes. It has a structure similar to that of P-gp, but a narrow range of substrates, which primarily consist of conjugated bile salts [112]. Efflux of bile salts by the BSEP is the rate-limiting step in bile production, and is essential for hepatocyte health [113]. The BSEP is not strictly a drug efflux transporter but it can be inhibited by common, clinically utilized drugs, such as rifampin [114], resulting in functional cholestasis and hepatocellular damage [115]. Thus, although the BSEP does not directly influence drug disposition or DDIs, its interaction with medications can be a cause of drug-induced liver injury and cholestasis. The degree to which this is important in equine medicine is not currently known because the role of the BSEP in horses, and its involvement in drug-induced liver injury has not been defined.

## 5. The Potential Role of Transporters at Blood Tissue Barriers in Horses

In addition to their functions in the liver, kidney, and small intestine, transporters play an important role in the blood–tissue barriers that protect privileged or pharmacological sanctuary sites. Organs, such as the brain and central nervous system (CNS), eye, prostate, testis, and placenta are protected from xenobiotic agents by highly specialized endothelial cell modifications in the capillaries that supply them. These cellular specializations prevent the movement into the tissues of the small lipophilic molecules that would normally be able to freely diffuse across the vascular endothelium.

The BBB is a barrier site of great clinical importance as it directly impacts the movement of drugs and toxins into the CNS. The endothelial cells of the capillaries supplying the brain are joined by tight junctions that profoundly limit paracellular movement of molecules. Efflux transporters are expressed on the luminal surface of the capillary endothelial cells to prevent the passage of drugs and other xeno- and endobiotics through the cells [3]. This strict gatekeeping is essential for normal neuronal function, but clinically impacts which drugs can be used to treat disorders of the CNS as many medications cannot gain entry to the brain. To circumvent the limitations imposed by the BBB, strategies such as coadministration of transporter inhibitors, osmotic or ultrasound disruption of the BBB, peptide-based delivery, or nanoparticle conjugation, have been pursued [116]. Selective use of technologies such as nanoparticle conjugation may allow not only delivery across the BBB, but also direct targeting of specific neural pathways within the brain [117].

The main efflux transport proteins expressed by the endothelial cells of the BBB are the ABC transporters P-gp and BCRP [3], and MRPs 1, 4, and 5 [107]. A recent study using mice, rats, and human cells found a lack of MATE protein expression in the BBB [118]. In veterinary medicine, many of the studies of BBB transporter expression have focused on the *Abcb1* (also known as *Mdr1*) gene polymorphism that occurs predominantly in herding dogs and results in toxicity after administration of normal doses of ivermectin and other P-gp substrates. A recent study quantified transporter expression in the canine brain and found that Bcrp was the most highly expressed efflux protein in the brain capillaries, followed by P-gp. Mrp1 and Mrp4 were below the limit of quantification of the LC-MS/MS technique used in that study, indicating extremely low or no protein expression [119]. However, in the choroid plexus the reverse was true, with expression of Mrp1 and Mrp4 dominating [119]. In the horse, most of the work that has been done on the BBB has been in relation to the pathogenesis of diseases caused by encephalitogenic viruses. However, certainly BBB transporter expression in the horse is relevant to veterinary medicine. Horses are susceptible to bacterial and viral encephalitis/meningitis as well as protozoal infestations of the CNS. They can also be affected by neurotoxins in plants, such as the toxins in *Oxytropis* and *Centaurea* species, which target the brain and CNS. They are highly susceptible to fumonisin, a toxin produced by *Fusarium* fungi that causes leukoencephalomalacia of the equine brain [120]. Horses have also suffered ivermectin-associated neurotoxicity; in one case, 11 of 15 horses developed symptoms of neurologic dysfunction after routine ivermectin treatment, and six were presented to an equine hospital for evaluation; investigation revealed that the horses were being fed hay contaminated with *Solanum* spp., which is thought to contain compounds that are P-gp inhibitors [121]. Better understanding of drug transporter expression in the equine BBB will allow improved therapy of equine CNS diseases and toxicities.

Other pharmacological sanctuaries include the eye [122], prostate [123], testis [124], and placenta [125]. These tissues all limit the access of xenobiotics through mechanisms similar to those of the BBB, including expression of efflux transporters; this protects them from potential toxicities but can make therapeutics more difficult. For example, many compounds do not cross the placental barrier [126], which is clinically helpful when treating a condition unrelated to the placenta or fetus, but can also reduce the efficacy of treatments aimed at placentitis or other reproductive conditions in the pregnant animal. In horses, the antimicrobial gentamicin has been shown to not cross the placenta [127], although the specific mechanism by which it is excluded has not been investigated. Very little work has been done to identify what other drugs do, or do not, cross the maternal blood–placental barrier in horses, and what drugs may be safely given to pregnant mares. Relying on results from human studies is unlikely to provide clinically accurate information; in part because of anatomical differences in placental anatomy that may impact barrier function, and also because of the high likelihood of species differences in transporter expression. Studies of ocular transporters in dogs, humans, and other species have highlighted both the similarities between species, and the differences. For instance, P-gp plays an important role in limiting the availability of topical ophthalmic medications administered to the cornea of rabbits but is less important in dogs [128]. Examples such as this highlight that species differences in drug transporter expression at blood–tissue barriers have practical and important clinical ramifications. Species-specific data are needed for rational, evidence-based therapeutic decision-making in veterinary medicine.

## 6. Conclusions

Since Juliano and Ling first described P-gp in 1976 [129], our understanding of the role of drug transporters has grown exponentially. In human medicine, detailed understanding of transporter expression and function has allowed DDIs to be avoided or exploited for clinical gain. Knowledge of transporter expression and activity is also critical for accurate PBPK modeling, and allows differences in transporter expression between animals and cell culture systems to be accounted for during in vitro to in vivo extrapolation of pharmacokinetic parameters [38]. These processes are critical to safe and effective development of pharmaceutical agents and protocols. However, veterinary medicine has lagged behind human medicine in developing a clinically relevant depth of understanding of drug transporter biology in domestic animal species. This is especially evident in the horse, a species for which there has been very little work on transporter function. Horses are both companion animals and economically valuable livestock, and the equine industry is economically important in terms of contribution to gross domestic product and job creation. This is reflected in the specialist training in equine medicine and surgery that is available to veterinarians. There are hospitals devoted only to horses, and procedures such as computed tomography, magnetic resonance imaging, fluoroscopy, and complex medical and surgical therapies can be provided to equine patients. It is time that we address the lack of data on equine drug disposition and begin to understand the specifics of drug transport in the horse. It is my hope that this review will stimulate interest in basic research on drug transporter function in horses and other veterinary species. It is time to stop guessing and start understanding our patients, of all species.

## Figures and Tables

**Table 1:** Drug transport proteins that have been experimentally identified in horses. P-gp = permeability glycoprotein (*Abcb1*); Bcrp = breast cancer resistance protein (*Abcg2*); Mrp1 = multidrug resistance protein 1 (*Abcc1)*; Mrp2 = multidrug resistance protein 2 (*Abcc2*); Oatp2b1 = organic anion transporting polypeptide 2b1 (*Slco2b1*). * Identified by mRNA expression only (all others were by protein expression). + Function in horses is assumed to be similar to function in humans; with the exception of p-glycoprotein, equine transporter function has not been experimentally determined.

Equine Transporter	NCBI Protein Accession #	Equine Tissue Distribution	Human Transporter (% Homology)	Function (Human) ^+^	Reference
P-gp	XP_014594657	Intestine (apical) Liver (apical) Kidney (apical primarily) Lymphocytes	P-gp (90%)	efflux	[34,78]
Bcrp	XP_005608692	Intestine (apical) Liver (apical, intracytoplasmic) Kidney (apical)	BCRP (86%)	efflux	[100]
Mrp1	NP_001075232	Intestine (intracytoplasmic)	MRP1 (90%)	efflux	[100]
Mrp2	XP_001500757	Intestine (apical) Liver (apical) Kidney (apical, some basolateral)	MRP2 (83%)	efflux	[100]
Oatp2b1 *	NP_001075258	Liver Kidney Brain Spleen Ovarian follicle Heart Skeletal muscle Testis Skin	OATP2B1 (80%)	uptake	[50]
